# In Vitro Evaluation of the Individual and Combined Cytotoxic and Estrogenic Effects of Zearalenone, Its Reduced Metabolites, Alternariol, and Genistein

**DOI:** 10.3390/ijms22126281

**Published:** 2021-06-11

**Authors:** Adrienn Balázs, Zelma Faisal, Rita Csepregi, Tamás Kőszegi, Balázs Kriszt, István Szabó, Miklós Poór

**Affiliations:** 1 Department of Environmental Toxicology, Institute of Aquaculture and Environmental Safety, Hungarian University of Agriculture and Life Sciences, Páter Károly u. 1, H-2100 Gödöllő, Hungary; balazs.adrienn@uni-mate.hu (A.B.); szabo.istvan.temi@uni-mate.hu (I.S.); 2 Department of Pharmacology, Faculty of Pharmacy, University of Pécs, Rókus u. 2, H-7624 Pécs, Hungary; faisal.zelma@gytk.pte.hu; 3 Food Biotechnology Research Group, János Szentágothai Research Centre, University of Pécs, Ifjúság útja 20, H-7624 Pécs, Hungary; koszegi.tamas@pte.hu; 4 Lab-on-a-Chip Research Group, János Szentágothai Research Centre, University of Pécs, Ifjúság útja 20, H-7624 Pécs, Hungary; ritacsepregi93@gmail.com; 5 Department of Laboratory Medicine, Medical School, University of Pécs, Ifjúság útja 13, H-7624 Pécs, Hungary; 6 Department of Environmental Safety, Institute of Aquaculture and Environmental Safety, Hungarian University of Agriculture and Life Sciences, Páter Károly u. 1, H-2100 Gödöllő, Hungary; kriszt.balazs@uni-mate.hu

**Keywords:** zearalenone, reduced zearalenone metabolites, alternariol, genistein, cytotoxicity, estrogenicity, combined effects, co-exposure

## Abstract

Mycotoxins are toxic metabolites of filamentous fungi. Previous studies demonstrated the co-occurrence of *Fusarium* and *Alternaria* toxins, including zearalenone (ZEN), ZEN metabolites, and alternariol (AOH). These xenoestrogenic mycotoxins appear in soy-based meals and dietary supplements, resulting in the co-exposure to ZEN and AOH with the phytoestrogen genistein (GEN). In this study, the cytotoxic and estrogenic effects of ZEN, reduced ZEN metabolites, AOH, and GEN are examined to evaluate their individual and combined impacts. Our results demonstrate that reduced ZEN metabolites, AOH, and GEN can aggravate ZEN-induced toxicity; in addition, the compounds tested exerted mostly synergism or additive combined effects regarding cytotoxicity and/or estrogenicity. Therefore, these observations underline the importance and the considerable risk of mycotoxin co-exposure and the combined effects of mycoestrogens with phytoestrogens.

## 1. Introduction

**Figure 1:**
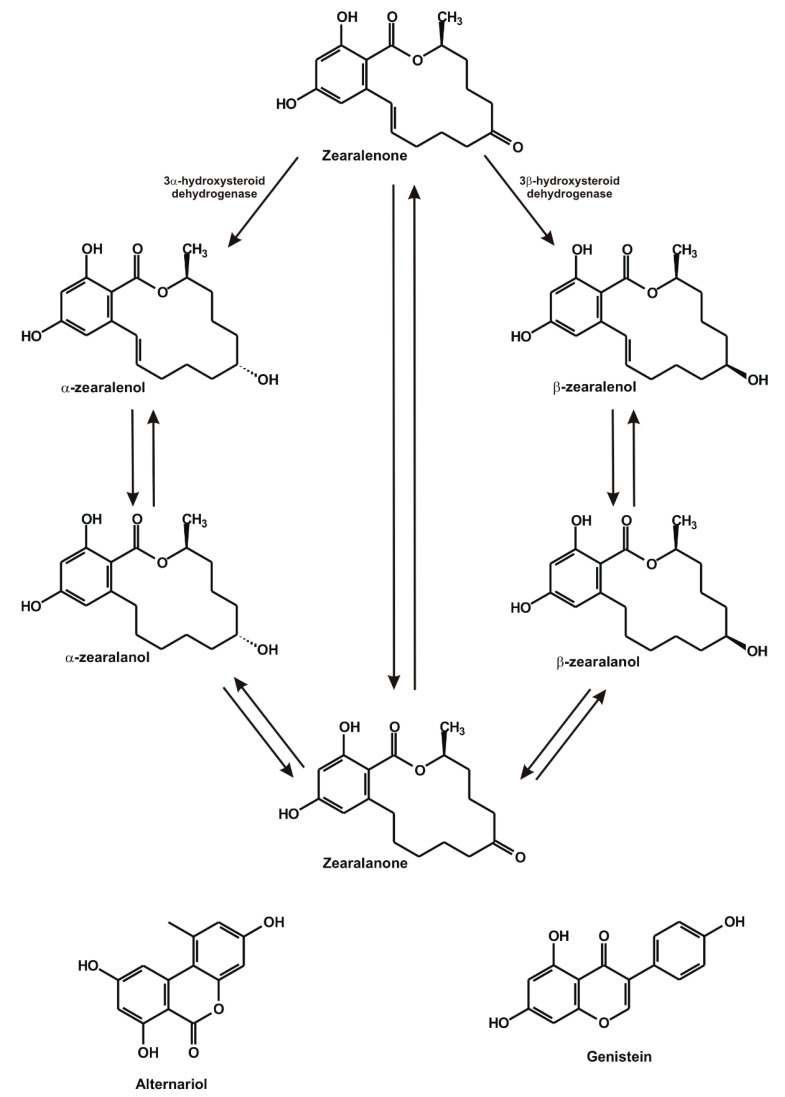
Chemical structures of zearalenone (ZEN), α-zearalenol (α-ZEL), β-zearalenol (β-ZEL), zearalanone (ZAN), α-zearalanol (α-ZAL), β-zearalanol (β-ZAL), alternariol (AOH), and genistein (GEN).

Mycotoxins, the toxic secondary metabolites of filamentous fungi, are common contaminants in food and animal feed [1]. Chronic exposure to mycotoxins poses a significant health risk for both animals and humans [2]. Zearalenone (ZEN; Figure 1) is a *Fusarium*-derived xenoestrogenic mycotoxin, which frequently occurs in grains (especially in maize) and in cereal-based products [3]. Despite its non-steroidal structure, ZEN can activate estrogen receptors causing reproductive disorders [4,5,6]. After its oral exposure, ZEN is extensively biotransformed via phase I and II reactions [4]. 

Hydroxysteroid dehydrogenase enzymes can reduce ZEN, resulting in the formation of α- and β-zearalenols (α- and β-ZELs, respectively; Figure 1) [3,4]. Furthermore, ZEN and ZELs can be further reduced to zearalanone (ZAN) and zearalanols (α- and β-ZALs), respectively [3,4]. Thereafter, ZEN and its reduced metabolites are subjected to glucuronide or sulfate conjugations [4]. Among the reduced ZEN metabolites, α-ZEL and α-ZAL exert considerably higher estrogenic activity than the parent mycotoxin [7,8,9]. In addition, α-ZAL is used as a growth promoter for animals in non-EU countries [4,10].

Alternariol (AOH; Figure 1) is a mycotoxin produced by *Alternaria* species [11]. It appears as a contaminant in cereals, seeds, rotten fruits, and vegetables (e.g., grapes and tomato) and the corresponding products (e.g., wine and tomato juice) [12]. The acute toxicity of AOH is considered to be low; however, the chronic exposure to AOH may be involved in the development of esophageal cancer [13]. Mutagenic and genotoxic effects have also been attributed to AOH [11,12]. The dibenzo-α-pyrone chemical structure of AOH shows similarity to certain estrogen-like compounds (e.g., urolithins and genistein); in addition, its androgenic/antiandrogenic effects also seem to be important regarding the endocrine disruptor effects of this mycotoxin [12,13,14,15,16].

Genistein (GEN; Figure 1) is a soybean isoflavone. Due to its structural similarity to 17β-estradiol, GEN can activate estrogen receptors and is considered as a phytoestrogen [17]. Therefore, numerous dietary supplements contain GEN, which are applied in the alternative treatment of perimenopausal, menopausal, and post-menopausal symptoms [17,18].

Previous studies highlighted the common co-occurrence of different filamentous fungi, such as *Fusarium* and *Alternaria* genera [19,20,21,22,23,24]. The simultaneous exposure to different mycotoxins and/or mycotoxin metabolites can cause combined impacts, such as antagonism, additive effects, or synergism [25]. Antagonism means that the combined impact is lower than the sum of the individual effects; an additive effect is evoked if the combined impact is close to the sum of the individual effects; while synergism can be observed when the combined impact is considerably higher than the sum of the individual effects [26]. 

Few in vitro studies have examined the combined cytotoxic effects of ZEN and ZELs: most of the data available suggest additive effects or synergism as the combined impacts of these mycotoxins [27,28,29,30]. The combined cytotoxic effects of ZEN with ZAN, ZALs, and AOH as well as the combined estrogenic effects of ZEN with its reduced metabolites (α-ZEL, β-ZEL, ZAN, α-ZAL, and β-ZAL) have not been previously examined. However, using in vitro models, synergism was observed regarding the co-treatment of ZEN with AOH [31] and ZEN with GEN [32]. 

Since the combined (e.g., additive and synergism) effects of mycotoxins pose a significant health risk, there is an urgent demand to explore the importance of mycotoxin-mycotoxin co-exposures, including mycotoxin metabolites. ZEN derivatives can be produced in humans and animals; nevertheless, ZELs and/or other metabolites have also been detected in certain cereals [33,34,35]. Furthermore, the presence of ZEN in soybeans [36,37] as well as the co-occurrence of ZEN and α-ZEL in soy food samples [38] have been described. Moreover, the simultaneous appearance of ZEN and AOH in certain soy-based dietary supplements has been reported [39], suggesting their co-exposure to GEN.

Considering the frequent co-occurrence of mycotoxins and mycotoxin metabolites, we examined the individual and combined effects of ZEN with ZELs, ZAN, ZALs, and AOH on the viability of HeLa cells. In addition, we evaluated the estrogenicity of these mycotoxins and GEN as well as the combined impacts of ZEN with reduced ZEN metabolites, AOH, and GEN based on a BLYES assay on *Saccharomyces cerevisiae*.

## 2. Results and Discussion

### 2.1. Individual Effects of ZEN, ZEN Metabolites, and AOH on Cell Viability

The cytotoxic effects of ZEN, its metabolites, and AOH were tested on a human cervical cancer cell line (HeLa). Based on previous investigations, these mycotoxins form highly stable complexes with serum albumin [40,41,42,43], which can strongly affect their cellular uptake and in vitro toxicity. Therefore, cell experiments were performed in the absence of FBS. Furthermore, the antiproliferative impacts of GEN vs. tumor/cancer cell lines have been reported in previous studies [44,45]; therefore, we examined the combined effects of ZEN and GEN only in the estrogenicity assay (see in Section 2.4).

**Figure 2:**
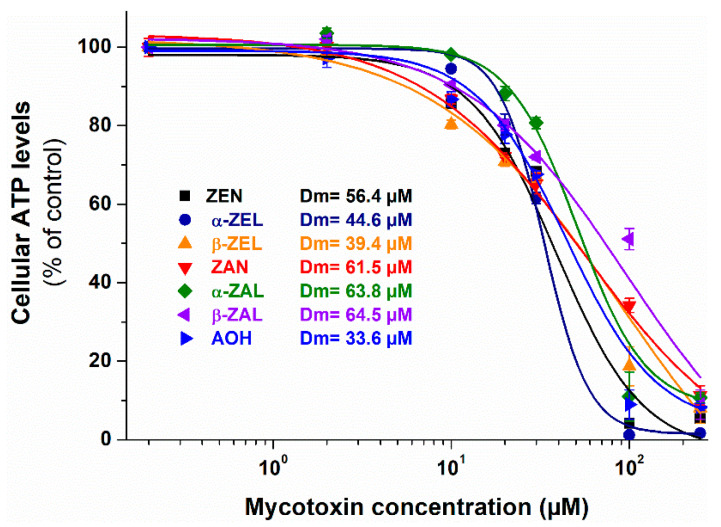
The effects of ZEN, α-ZEL, β-ZEL, ZAN, α-ZAL, β-ZAL, and AOH on the viability of HeLa cells (ATP/well, % of control) after 24 h incubation. Statistically significant (*p* < 0.01) decreases in cell viability were noticed at (and above) the following mycotoxin concentrations: 10 µM for AOH, ZEN, ZAN, β-ZEL, and β-ZAL; and 20 µM for α-ZEL and α-ZAL.

The cytotoxicity of mycotoxins was evaluated based on the ATP levels/well [46]. In a concentration-dependent fashion, the mycotoxins tested induced a gradual decrease in ATP levels (Figure 2). In agreement with previous studies, the acute toxicity of these compounds was relatively low [47,48,49]. AOH showed the highest toxicity, while ZAN and ZALs demonstrated the lowest impacts on cell viability (Figure 2); nevertheless, the acute cytotoxic effects of the compounds tested can be considered similar (IC_50_ or *D_m_* = 34 to 65 µM).

The in vitro cytotoxicity of ZEN, α-ZEL, and β-ZEL was also tested on HeLa cells in another study after 24 h incubation (RPMI-1640 medium, with 10% fetal calf serum) [47]. They noticed similar effects of ZEN and α-ZEL, while β-ZEL showed a less toxic impact. However, in our investigation, only slight differences were observed in the cytotoxicity of ZEN and ZELs (Figure 2), which can likely be explained by the different cell culture medium applied (DMEM without FBS in the current study).

The in vitro cytotoxic effects of ZEN and/or its metabolites have been studied in several cell cultures; nevertheless, the reported data are controversial. ZEN was more toxic than ZELs on Caco-2 (human colorectal adenocarcinoma) [50], HeLa (human cervical adenocarcinoma) [47], and swine neutrophil [48], as well as on KB (human oral epidermoid carcinoma) and MCF-7 (human breast adenocarcinoma) [49] cells. In contrast, the higher cytotoxicity of ZELs vs. ZEN has been reported on CHO-K1 (Chinese hamster ovary) [27] and H460 (human non-small cell lung carcinoma) [51] cell lines. 

Furthermore, most of the earlier studies suggested the higher cytotoxicity of β-ZEL compared to α-ZEL, which have been demonstrated on Caco-2 [50], Vero (monkey kidney cells) [52], RAW264.7 macrophages (Abelson murine leukemia virus-induced tumor) [53], SH-SY5Y (human neuroblastoma) [54], and HepG2 (human hepatocellular carcinoma) [28] cells. On the other hand, some studies demonstrated that α-ZEL was more cytotoxic than β-ZEL on CHO-K1 [27] and HeLa [47] cell lines; while similar impacts of ZELs have been described in swine neutrophils [48] and Vero cells [49].

Less data are available regarding the comparison of ZAN and ZALs. ZAN was less toxic than ZEN on Vero cells [49] and swine neutrophils [48]. The lower cytotoxicity of α-ZAL vs. ZEN has been noticed on KB, MCF-7, and Vero cells [49]. Furthermore, β-ZAL was more toxic on H460 [51] and Vero cells [49], while it showed lower cytotoxicity on KB and MCF-7 cell lines [49] compared to ZEN. In a recent study, AOH induced a higher cytotoxic effect than ZEN on Ishikawa cells (human endometrial cancer cell line) [55], which agrees with our results (Figure 2). Furthermore, the cytotoxicity of AOH has also been demonstrated on HeLa [56] and HepG2 [57] cell lines.

Our results and the above-listed reports underline that the experimental conditions (the cell lines used, cell culture media applied, etc.) can considerably affect the cytotoxicity induced by ZEN, reduced ZEN metabolites, and AOH.

### 2.2. Combined Effects of Mycotoxins on Cell Viability

**Figure 3:**
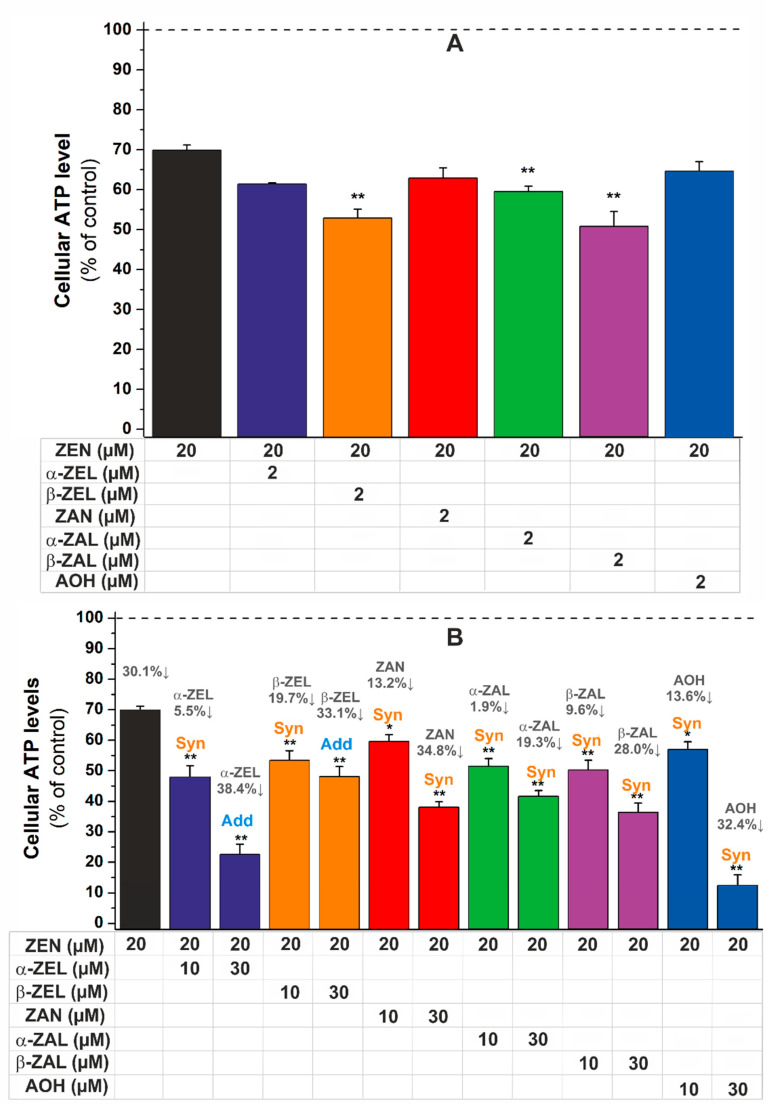
The effects of ZEN alone and in combination with the subtoxic (**A**) or toxic (**B**) concentrations of α-ZEL, β-ZEL, ZAN, α-ZAL, β-ZAL, and AOH on HeLa cells after 24 h incubation (ATP/well, % of control; changes compared to the ZEN-exposed cells: * *p* < 0.05 and ** *p* < 0.01). The individual effects of mycotoxins at the concentrations applied are demonstrated with dark grey color (**B**).

As the co-occurrence of mycotoxins and their metabolites is common in food and feed [19,20,21,22,23,24], the combined cytotoxic effects of ZEN with ZELs, ZAN, ZALs, and AOH were also investigated with HeLa cells. First, the co-exposure to the toxic concentration of ZEN and the subtoxic concentrations of its metabolites and AOH was tested. Even at their non-toxic concentrations (Figure 2), β-ZEL, α-ZAL, and β-ZAL induced statistically significant (*p* < 0.01) decreases in ATP levels (Figure 3A). This is interesting because ZALs were the least toxic derivatives in the single compound tests (Figure 2).

The co-treatment of the same amount of ZEN with two toxic concentrations of other mycotoxins was also examined. In a concentration-dependent fashion, reduced ZEN derivatives and AOH induced a significant decrease in cell viability (Figure 3B). The combined effects were evaluated with the CompuSyn software (see details in 3.4). According to the combination index (*CI*) values calculated, synergism was observed in the presence of ZAN, ZALs, and AOH at both concentrations applied (Figure 3B). The lower concentrations of ZELs also resulted in synergism, while their higher concentrations showed additive effects with the parent mycotoxin (Table 1).

In a previous study, the co-treatment of 30 µM ZEN with 15 or 30 µM α-ZEL showed antagonism on HepG2 (48-h incubation) cells; however, the combination of 60 µM ZEN with 15 or 30 µM α-ZEL resulted in synergism based on the MTT cytotoxicity assay [29]. In another report, the combined cytotoxic effects of ZEN and ZELs was tested on HepG2 cells (72-h treatment) using a neutral red assay: synergism was described regarding the combinative impacts of ZEN and α-ZEL (1:1 molar ratio) regardless of the mycotoxin concentrations applied; and the co-exposure to ZEN and β-ZEL showed additive effects or synergism [28]. 

In contrast, the combined cytotoxic (MTT assay) effects of ZEN with α-ZEL showed antagonism on HepG2 cells after 24- and 72-h incubations; while the 48-h treatment represented controversial impacts (synergism, additive effects, or antagonism) depending on the concentrations used [30]. The combinations of ZEN with α- or β-ZELs were also tested on a CHO-K1 cell line (24-, 48-, and 72-h treatments) applying a MTT viability assay, where mostly additive impacts (or synergism) were reported [27].

Based on our results and the above-listed studies [27,28,29,30], the experimental conditions used (e.g., cell line, concentrations, and incubation time) can strongly affect the combined effects of ZEN and ZELs. However, most of the studies available suggest additive effects or synergism as the combined impacts of these mycotoxins. Based on our knowledge, there are no previous investigations regarding the combined cytotoxic effects of ZEN with ZAN, ZALs, and AOH. Nevertheless, our results underline that even the subtoxic concentrations of certain ZEN metabolites can increase the toxicity of ZEN, and synergism can be typically observed during the co-treatment of the parent mycotoxin with its reduced derivatives or AOH.

### 2.3. Individual Estrogenic Effects of ZEN, ZEN Metabolites, AOH, and GEN

**Figure 4:**
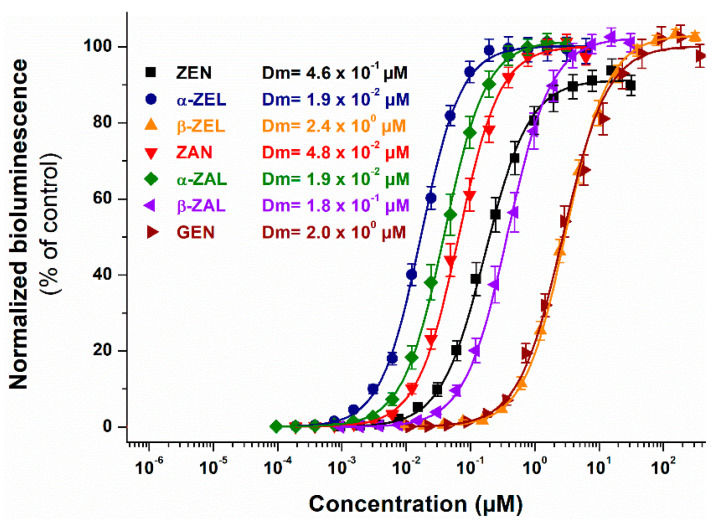
Estrogenic effects of ZEN, α-ZEL, β-ZEL, ZAN, α-ZAL, β-ZAL, and GEN based on the BLYES (*Saccharomyces cerevisiae*) assay. Statistically significant (*p* < 0.01) estrogenic effects were observed at (and above) the following mycotoxin concentrations: 3.1 × 10^−2^ µM for ZEN, 1.5 × 10^−3^ µM for α-ZEL, 3.1 × 10^−1^ µM for β-ZEL, 1.2 × 10^−2^ µM for ZAN, 2.4 × 10^−2^ µM for α-ZAL, 3.0 × 10^−2^ µM for β-ZAL, and 7.2 × 10^−1^ µM for GEN.

In the BLYES assay, each compound tested produced full concentration-response curves (Figure 4), except AOH, which did not induce estrogenic effects in this model even at a 50 µM concentration (data not shown). Since most of these molecules possess strong xenoestrogenic effects, generally their low concentrations were applied. Nevertheless, to produce the appropriate sigmoidal curves for evaluation, higher than 3 µM concentrations of ZEN, β-ZEL, β-ZAL, and GEN were also used. Importantly, these concentrations induced the maximal response in the BLYES assay (Figure 4). Regarding AOH, no significant bioluminescence induction/reduction was observed in comparison to the solvent control at any concentrations tested (data not shown).

In the BLYES assay, α-ZEL and α-ZAL exhibited the strongest estrogenicity followed by ZAN, β-ZAL, and ZEN according to the *D_m_* (EC_50_) values. The less potent compounds were GEN and β-ZEL (Figure 4). In agreement with our observations, α-ZEL, α-ZAL, ZAN, and β-ZAL showed stronger effects, whereas β-ZEL produced weaker estrogenic effects compared to ZEN in earlier studies [6,58]. Other in vitro reports also demonstrated that, among ZEN metabolites, α-ZEL was the most potent xenoestrogen [4,59,60].

The phytoestrogen GEN showed very similar estrogenic activity in the BLYES assay as β-ZEL (Figure 4). Furthermore, the BLYES strain represented similar sensitivity to GEN as the recombinant yeast expressing the hERα and lacZ genes [61]. However, in the MCF-7 cell proliferation assay, the EC_50_ value of GEN was two orders of magnitude lower [62] than in the BLYES assay. Based on previous studies, the diaryl ring and hydroxyl substitution are indispensable for the estrogenic activity of flavonoids [63]. GEN binds to hERβ with 20-fold higher affinity than to hERα, and the lower binding affinity of GEN (vs. ZEN) toward hERα was previously reported [64]. These data and the fact that *S. cerevisiae* BLYES strain harbors the hERα gene on its chromosome [65] explain the lower estrogenicity of GEN in the BLYES assay.

The weak estrogenic activity of AOH has been reported in some studies; however, AOH did not show estrogenicity in the BLYES assay. A previous study suggested 10,000- and 2500-fold lower affinity of AOH toward hERα and hERβ compared to 17β-estradiol (E2), respectively [15]. Furthermore, the estrogen-dependent alkaline phosphatase assay (on Ishikawa cells) demonstrated that AOH has 10,000-fold weaker estrogenic effects vs. E2 [15]. In another study (yeast reporters bioassay), AOH behaved as a weak partial agonist of estrogen and as a full agonist of androgen receptors [16]. On the other hand, antiandrogenic effects of lower AOH concentrations have been described [16]. Further mechanisms may also take part in the endocrine disruptor effects of AOH, as the mycotoxin significantly increased estrogen production in H295R (human adrenocortical carcinoma) cells, due to the modulation of the steroidogenesis pathway [66].

### 2.4. Combined Estrogenic Effects of ZEN, ZEN Metabolites, AOH, and GEN

**Figure 5:**
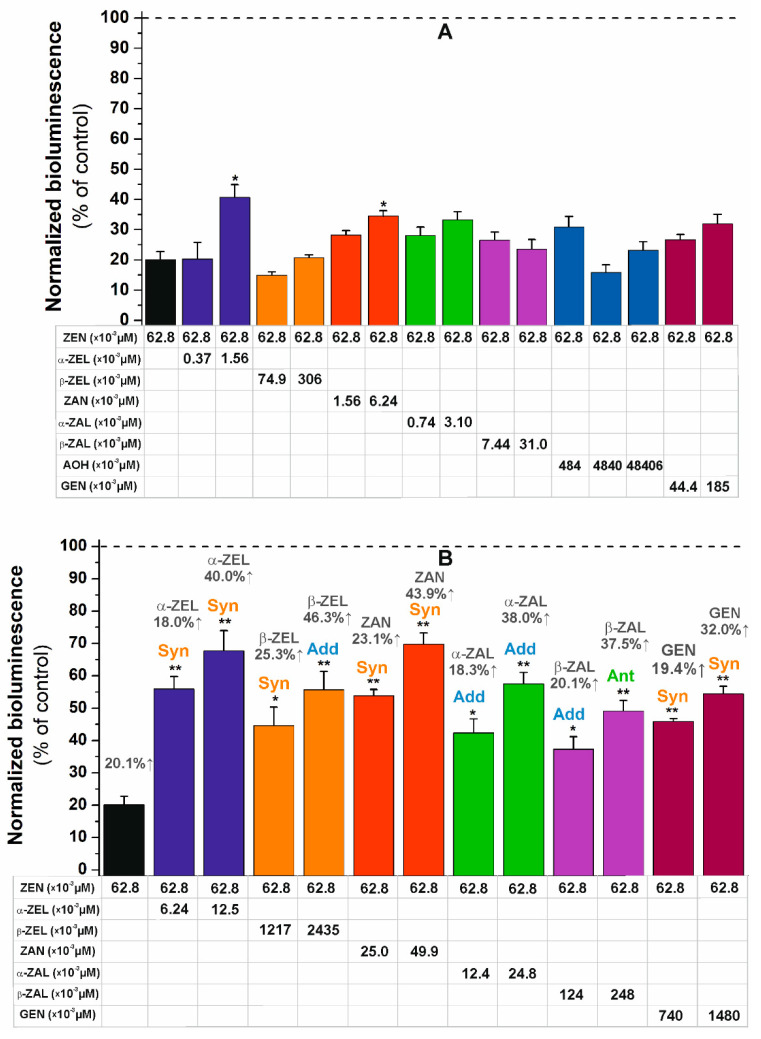
Estrogenic effects of ZEN alone and in combination with the subtoxic (non-estrogenic; (**A**)) or toxic (estrogenic; (**B**)) concentrations of α-ZEL, β-ZEL, ZAN, α-ZAL, β-ZAL, AOH, and GEN based on the BLYES (*Saccharomyces cerevisiae*) assay (* *p* < 0.05 and ** *p* < 0.01). The individual effects of mycotoxins at the concentrations applied are demonstrated with a dark grey color (**B**).

First, the toxic concentration of ZEN (showing approximately 20% estrogenicity in the model applied) was combined with the subtoxic (no significant estrogenicity) concentrations of ZELs, ZAN, ZALs, AOH, and GEN. Compared to the individual effects of ZEN, only the higher subtoxic concentrations of α-ZEL (1.56 × 10^−3^ µM) and ZAN (6.24 × 10^−3^ µM) induced statistically significant (*p* < 0.05) increases in the estrogenic effect of the parent mycotoxin (Figure 5A). AOH did not cause any relevant change in the estrogenicity of ZEN, even at the highest concentration used (48.4 µM; Figure 5A).

In the following experiment, the same amount of ZEN was combined with the estrogenic concentrations of ZEN metabolites and GEN. In a concentration-dependent fashion, each compound caused a significant increase in estrogenicity (Figure 5B). Based on the evaluation with the CompuSyn software (see details in Section 3.4), synergism was observed in the presence of α-ZEL, ZAN, and GEN at both concentrations tested (Table 2). Furthermore, β-ZEL showed synergism and additive effects in its lower and higher concentrations tested, respectively. The additive combined effects of ZEN and α-ZAL were noticed; while β-ZAL demonstrated additive effect at its lower concentration but showed antagonism at its higher toxic concentration examined (Figure 5B, Table 2).

To the best of our knowledge, this is the first study to evaluate the combined estrogenic effects of ZEN with its reduced metabolites (α-ZEL, β-ZEL, ZAN, α-ZAL, and β-ZAL). Interestingly, ZELs and ZAN mainly showed synergism, whereas ZALs mostly triggered additive combined estrogenic effects with ZEN (Figure 5). 

Only a few studies investigated the estrogenic combined effects of ZEN with AOH or GEN. In a previous study, the impacts of AOH (50 nM to 10 µM) in combination with ZEN (10 pM to 10 µM) and α-ZEL (1 pM to 10 µM) were examined [31]. Each compound showed estrogenic activity; in addition, ZEN and α-ZEL increased the effect of AOH (synergism). A significant induction in alkaline phosphatase activity was observed in Ishikawa cells as a result of the combined treatment with low concentrations of AOH (5 µM) and ZEN (100 nM) [31]. In contrast, in the current study, even the high concentrations of AOH did not increase the estrogenic effect of ZEN (Figure 5A). The different results can be explained by the distinct experimental models used: the BLYES strain contains only ERα [65], whereas Ishikawa cells express both ERα and ERβ [67]. Importantly, AOH has a higher affinity toward ERβ [15], whereas ZEN prefers ERα [64].

In another study, the combined estrogenic effects of ZEN and GEN were also tested, based on the estrogen dependent activation of alkaline phosphatase on Ishikawa cells [32]. These data are in agreement with our results (Figure 5), synergism was observed as the combined estrogenic effects of GEN and ZEN. Therefore, these data also highlight the risk regarding the co-exposure to mycoestrogens and phytoestrogens.

## 3. Materials and Methods

### 3.1. Reagents

Zearalenone (ZEN), α-zearalenol (α-ZEL), β-zearalenol (β-ZEL), zearalanone (ZAN), α-zearalanol (α-ZAL), β-zearalanol (β-ZAL), genistein (GEN), Dulbecco’s Modified Eagle’s Medium (DMEM), and 17β-estradiol (E2) were purchased from Sigma-Aldrich (St. Louis, MO, USA). Alternariol (AOH) was obtained from Cfm Oskar Tropitzsch (Marktredwitz, Germany). Bioluminescent ATP Assay Kit CLSII (Roche; Basel, Switzerland) and fetal bovine serum (FBS; Pan-Biotech; Aidenbach, Germany) were used as received. Methanol (synthesis grade) was purchased from Reanal Laboratory Chemicals Ltd. (Budapest, Hungary). ZEN applied in the BLYES assay was obtained from Fermentek (Jerusalem, Israel).

### 3.2. Cell Culturing, Treatment, and Cell Viability Assay

The HeLa cervical cancer adherent cell line (ATCC: CCL-2) was applied for the evaluation of individual and combined effects of mycotoxins on cell viability. The cells were cultured in DMEM (high glucose, 4500 mg/L) with 10% FBS, penicillin (100 U/mL), and streptomycin (100 μg/mL) in sterile plastic cell culture flasks (75 cm^2^; 5% CO_2_, 37 °C). The cells were trypsinized and plated onto 96-well sterile plastic plates. Stock solutions (each 5000 μM) of ZEN, ZELs, ZAN, and ZALs used in cell experiments were prepared in 96 *v*/*v*% ethanol (spectroscopic grade; VWR, Debrecen, Hungary). Stock solutions of AOH (5000 μM or 40 mM) were prepared in spectroscopic grade dimethyl sulfoxide (DMSO; Fluka, Morris Plains, NJ, USA). Mycotoxin stock solutions were stored at −20 °C. Before the treatment, the cell culture medium was replaced with a fresh one without FBS, because of the strong interactions of ZEN, ZEN metabolites, and AOH with serum albumin [40,41,42].

HeLa cells were treated with ZEN, α/β-ZEL, ZAN, α/β-ZAL, and AOH (0–250 μM) for 24 h. Since higher solvent concentrations can affect the cell viability, solvent controls were applied in each experiment. The highest DMSO (solvent of AOH) level used was 0.6 *v*/*v*%, which did not significantly decrease the ATP concentrations (96.8 ± 3.5% compared to the control). In most of the experiments, ethanol (solvent of ZEN and its metabolites) did not exceed 1 *v*/*v*%, which also barely modified the cellular ATP levels (96.3 ± 2.1% compared to the control). However, to produce a remarkable loss in cell viability, 100 and 250 μM (Figure 2) of mycotoxins were also employed in the single compound tests, where the final concentrations of ethanol were 2 and 5 *v*/*v*%, respectively. Since the ATP levels were affected by the presence of 2 *v*/*v*% (ATP concentrations: 94.0 ± 1.6% compared to the control) and 5 *v*/*v*% (ATP concentrations: 71.1 ± 4.2% compared to the control) ethanol, in these samples, the relative viability loss was calculated compared to the corresponding solvent controls. The cell viability was evaluated based on the ATP levels/well employing a previously described method [46] without modifications. The viability data were derived from cell experiments performed on different experimental days, and the mycotoxin-induced relative changes in cell viability showed good correlation (SEM < 6.3% in single compound experiments; and SEM < 3.8% in co-treatment experiments).

To test the combined effects of the mycotoxins, ZEN was used at one fixed concentration (that caused an approximately 30% decrease in cell viability in the single compound tests: 20 μM). ZELs, ZAN, ZALs, and AOH were added at 2 μM (subtoxic), 10 μM (an approximately 5–20% decrease in cell viability), and 20 μM (an approximately 10–40% decrease in cell viability) concentrations.

### 3.3. BLYES Assay in Saccharomyces Cerevisiae

The *Saccharomyces cerevisiae* BLYES strain was applied to examine the estrogenic activity of the compounds tested. This bioluminescent yeast bioreporter harbors the *hERα* gene on its chromosome, and it has two plasmids with genes responsible for bioluminescence. The pUTK404 plasmid contains *luxC, -D,* and *-E* genes from *Photorhabdus luminescens* and flavin oxidoreductase gene (*frp*) from *Vibrio harveyi*. The pUTK407 plasmid contains tandem estrogen responsive elements (EREs) that initiate the transcription of *luxA* and *luxB* genes after binding the dimer formed by estrogen receptor—ligand complexes [65].

To examine the combined effects, ZEN was used at one fixed concentration (that caused an approximately 20% effect in the single compound tests: 6.3 × 10^−2^ µM); furthermore, ZELs, ZAN, ZALs, or GEN were added at two subtoxic (the highest concentration that induced less than a 5% effect as well as its one-quarter) and two toxic (the concentrations that produced approximately 20% and 40% impacts) concentrations. Since AOH did not show relevant effects in the BLYES assay, it was applied only at subtoxic (the highest water soluble concentration as well as its tenth and its hundredth) concentrations.

*S. cerevisiae* BLYES strain was grown in 30 mL of yeast growth medium (YMM leu-, ura-) for 18–20 h at 30 °C. For preparing 1000 mL YMM (leu-, ura-), 854.5 mL yeast base medium (13.61 g KH_2_PO_4_, 1.98 g (NH_4_)_2_SO_4_, 4.2 g KOH, 0.2 g MgSO_4_, 1 mL FeSO_4_ solution (40 mg/50 mL water), 50 mg L-histidine, 50 mg adenine, 20 mg L-arginine-HCl, 20 mg L-methionine, 30 mg L-tyrosine, 30 mg L-isoleucine, 30 mg L-lysine-HCl, 25 mg L-phenylalanine, 100 mg L-glutamic acid, 150 mg L-valine, and 375 mg L-serine in 1000 mL distilled water) was supplemented with 100 mL glucose solution (20 *w*/*v*%), 25 mL L-aspartic acid (4 mg/mL), 8 mL L-threonine (24 mg/mL), 10 mL vitamin solution (8 mg thiamine, 8 mg pyridoxine, 8 mg pantothenic acid, 40 mg inositol, 20 mL biotin (2 mg/100 mL) in 180 mL distilled water), and 2.5 mL CuSO_4_ × 5H_2_O (0.3745 g/50 mL) solutions [61].

Stock solutions of the compounds tested were prepared in methanol and stored at −20 °C. Estrogen assays were carried out on 96-well (PS, F-bottom) microplates (Greiner Bio-One Hungary Ltd., Mosonmagyaróvár, Hungary). In single compound tests, stock solutions of the compounds were serially diluted with methanol; whereas, in co-exposure experiments, a 20-µL mixture of the solutions was placed into the wells of the microplates. After the evaporation of methanol, yeast culture (200 µL; OD600 of 1.000 ± 0.050) were pipetted into each well. E2 was applied as a positive control, while wells containing only yeast cells and yeast cells with methanol served as negative and solvent controls, respectively. Bioluminescence (cps, counts per second) was measured after 5-h incubation (30 °C) with a VictorX Multilabel Plate Reader (Perkin Elmer Inc., Waltham, MA, USA) [65].

BLYES assays were performed in more different experimental days, the mycotoxin-induced relative estrogenicity data showed good correlation (SEM < 4.5% in single compound experiments; and SEM < 6.2% in co-treatment experiments).

### 3.4. Assessment of Combined Effects

Combined effects were evaluated based on combination indices (*CI*) determined using the CompuSyn software (ComboSyn Inc., Paramus, NJ, USA) by the median-effect equation of the mass-action law [68]:

(1) $$log\frac{f_{a}}{f_{u}}=m\times \log\left(D\right)-m\times log(D_{m}),$$

where *fa* and *fu* are the affected and unaffected fractions of the system, respectively; *D* represents the dose, *D_m_* denotes the dose necessary for the production of median effect, while *m* is the sigmoidicity coefficient (*m* > 1 sigmoidal, *m* = 1 hyperbolic, *m* < 1 flat sigmoidal).

The combined effects of mycotoxins were determined by the *CI* values calculated based on the following equation [68]:

(2) $$CI=\frac{{\left(D\right)}_{1}}{{\left(D_{x}\right)}_{1}}+\frac{{\left(D\right)}_{2}}{{\left(D_{x}\right)}_{2}},$$

where (*D_x_*)_1_ and (*D_x_*)_2_ are the individual doses necessary for the production of *x*% effect, while (*D*)_1_ and (*D*)_2_ are the doses of the two compounds that combined evoke the same effect as (*D_x_*)_1_ alone or (*D_x_*)_2_ alone, i.e., the targeted x% effect. If the *CI* value is approximately 1 (0.8 < *CI* < 1.2), then the combination has an additive effect; while *CI* < 0.8 or *CI* > 1.2 indicate synergism or antagonism, respectively.

### 3.5. Statistics

The mean and standard error of the mean (±SEM) values were derived at least from three independent experiments. Statistical evaluation (*p* < 0.05 and *p* < 0.01) was performed using one-way ANOVA and Dunnett’s post hoc test (IBM SPSS Statistics, Armonk, NY, USA).

The bioluminescence values obtained in the BLYES assay were normalized between 0 and 100% applying GraphPad Prism 5.03 program (GraphPad Software Inc., San Diego, CA, USA). Zero percent was defined in each measurement as the mean of the methanol control values, and 100% was defined as the mean of all plateau values of single compounds’ concentration—response curves.

## 4. Conclusions

In the current study, the individual and combined effects of ZEN, reduced ZEN metabolites, AOH, and GEN were examined from the cytotoxicity and estrogenicity points of view. The EFSA Panel on Contaminants in the Food Chain (CONTAM) set the group tolerable daily intake (TDI) value of ZEN and its modified forms to 0.25 μg/kg body weight per day [69]. Despite the frequent co-occurrence of mycotoxins (and mycotoxin metabolites), risk assessment can evaluate the exposure to single mycotoxins, due to the limited data regarding the combined effects of ZEN, its metabolites, other mycotoxins, and/or further xenoestrogens/phytoestrogens. 

Our results demonstrate that the combined cytotoxic or estrogenic effects of ZEN with its metabolites, AOH, or GEN, were commonly additive or synergistic as can be observed (Table 1 and Table 2). Moreover, even the subtoxic concentrations of certain ZEN metabolites could aggravate the toxic effects of ZEN (Figure 3A and Figure 5A). Although our in vitro studies cannot be directly extrapolated to humans or animals, this investigation underlines the importance and the significant risk of combined mycotoxin effects and exposure.

## Figures and Tables

**Table 1:** The combination index values (*CI* < 0.8: synergism; 0.8 < *CI* < 1.2: additive effect; and *CI* > 1.2: antagonism) and combined cytotoxic effects of ZEN with cytotoxic concentrations of ZELs, ZAN, ZALs, and AOH.

20 µM ZEN +	*CI* Value	Combined Cytotoxic Effect	
10 µM α-ZEL	0.57	Synergism	++
30 µM α-ZEL	0.81	Additive effect	±
10 µM β-ZEL	0.64	Synergism	+
30 µM β-ZEL	1.07	Additive effect	±
10 µM ZAN	0.55	Synergism	++
30 µM ZAN	0.77	Synergism	+
10 µM α-ZAL	0.52	Synergism	++
30 µM α-ZAL	0.78	Synergism	+
10 µM β-ZAL	0.51	Synergism	++
30 µM β-ZAL	0.72	Synergism	+
10 µM AOH	0.73	Synergism	+
30 µM AOH	0.50	Synergism	++

ZEN, zearalenone; α-ZEL, α-zearalenol; β-ZEL, β-zearalenol; ZAN, zearalanone; α-ZAL, α-zearalanol; β-ZAL, β-zearalanol; AOH, alternariol; *CI* < 0.60: ++; 0.60 < *CI* < 0.80: +; and 0.80 < *CI* < 1.20: ±.

**Table 2:** The combination index values (*CI* < 0.8: synergism; 0.8 < *CI* < 1.2: additive effect; and *CI* > 1.2: antagonism), and combined estrogenic effects of ZEN with ZELs, ZAN, ZALs, and GEN.

6.28 × 10^−2^ µM ZEN +	*CI* Value	Combined Estrogenic Effect	
6.24 × 10^−3^ µM α-ZEL	0.37	Synergism	++
1.25 × 10^−2^ µM α-ZEL	0.41	Synergism	++
1.22 µM β-ZEL	0.77	Synergism	+
2.43 µM β-ZEL	0.95	Additive effect	±
2.50 × 10^−2^ µM ZAN	0.58	Synergism	++
4.99 × 10^−2^ µM ZAN	0.62	Synergism	+
1.24 × 10^−2^ µM α-ZAL	0.97	Additive effect	±
2.48 × 10^−2^ µM α-ZAL	1.16	Additive effect	±
1.24 × 10^−1^ µM β-ZAL	1.19	Additive effect	±
2.48 × 10^−1^ µM β-ZAL	1.56	Antagonism	--
7.4 × 10^−1^ µM GEN	0.58	Synergism	++
1.48 µM GEN	0.77	Synergism	+

ZEN, zearalenone; α-ZEL, α-zearalenol; β-ZEL, β-zearalenol; ZAN, zearalanone; α-ZAL, α-zearalanol; β-ZAL, β-zearalanol; GEN, genistein; *CI* < 0.60: ++; 0.60 < *CI* < 0.80: +; 0.80 < *CI* < 1.20: ±; 1.20 < *CI* < 1.40: -; and *CI* > 1.40: --.

## Data Availability

Not applicable.
